# Cardiocerebral and cardiopulmonary resuscitation – 2017 update

**DOI:** 10.1002/ams2.281

**Published:** 2017-05-26

**Authors:** Gordon A. Ewy

**Affiliations:** ^1^ Department of Medicine (Cardiology) University of Arizona College of Medicine Tucson AZ USA

**Keywords:** Bystander CPR, out‐of‐hospital cardiac arrest, primary cardiac arrest, respiratory arrest

## Abstract

Sudden cardiac arrest is a major public health problem in the industrialized nations of the world. Yet, in spite of recurrent updates of the guidelines for cardiopulmonary resuscitation and emergency cardiac care, many areas have suboptimal survival rates. Cardiocerebral resuscitation, a non‐guidelines approach to therapy of primary cardiac arrest based on our animal research, was instituted in Tucson (AZ, USA) in 2002 and subsequently adopted in other areas of the USA. Survival rates of patients with primary cardiac arrest and a shockable rhythm significantly improved wherever it was adopted. Cardiocerebral resuscitation has three components: the community, the pre‐hospital, and the hospital. The community component emphasizes bystander recognition and chest compression only resuscitation. Its pre‐hospital or emergency medical services component emphasizes: (i) urgent initiation of 200 uninterrupted chest compressions before and after each indicated single defibrillation shock, (ii) delayed endotracheal intubation in favor of passive delivery of oxygen by a non‐rebreather mask, (iii) early adrenaline administration. The hospital component was added later. The national and international guidelines for cardiopulmonary resuscitation and emergency medical services are still not optimal, for several reasons, including the fact that they continue to recommend the same approach for two entirely different etiologies of cardiac arrest: primary cardiac arrest, often caused by ventricular fibrillation, where the arterial blood oxygenation is little changed at the time of the arrest, and secondary cardiac arrest from severe respiratory insufficiency, where the arterial blood is severely desaturated at the time of cardiac arrest. These different etiologies need different approaches to therapy.

## Introduction

Cardiovascular diseases continue to be the leading cause of death in most industrialized nations of the world.^1^, ^2^ Unfortunately, the first sign of cardiovascular disease is often the last, as the first sign is often sudden cardiac arrest. In the USA, the average age of men with sudden cardiac arrest is their mid‐60s. In Japan, it is in their mid‐70s. A 40‐year‐old American man has a one in eight chance of having sudden cardiac arrest during his lifetime.^3^, ^4^ It is probably similar in other industrialized countries of the world.

**Figure 1:**
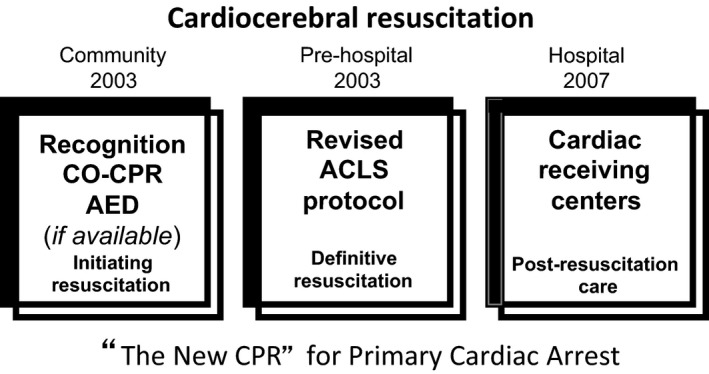
Three components of cardiocerebral resuscitation for primary out‐of‐hospital cardiac arrest secondary to a shockable rhythm. In 2003, the community and prehospital components were initiated in Arizona (USA). In 2007, the hospital component was added. ACLS, advanced cardiac life support; AED, automated external defibrillator; CO‐CPR, chest compression only cardiopulmonary resuscitation.

Accordingly, cardiac arrest is a major public health problem. Because of its different etiologies, cardiac arrest should be classified into two different categories. The vast majority is due to primary cardiac arrest. Secondary cardiac arrest, from respiratory insufficiency (such as drowning or drug overdose) is less common but still a major public health problem. Therefore, optimal therapy should be tailored to the etiology of the cardiac arrest: cardiocerebral resuscitation (CCR) for primary cardiac arrest (Fig. 1) and cardiopulmonary resuscitation (CPR) for secondary cardiac arrest.

The principal determinants of survival of individuals with witnessed primary cardiac arrest and a shockable rhythm, the subset most likely to survive, are a bystander's prompt recognition and appropriate response, early optimal therapy by emergency medical services personnel (EMS), and appropriate post‐resuscitation hospital care.

## Difference approaches for primary and secondary cardiac arrests are critically important

A major reason for different therapeutic approaches to resuscitation for these two distinct causes of cardiac arrest is the difference in the patient's arterial blood oxygenation at the onset of the arrests. In primary cardiac arrest, the blood oxygenation is generally normal at the time of the arrest and as forward blood flow quickly slows and stops, the arterial blood oxygenation remains normal for several minutes.^5^ In contrast, with drowning, drug overdose, or other causes of respiratory insufficiency, the heart continues to circulate blood for some time, but because of the lack of ventilation the arterial blood becomes progressively more desaturated, turning dark; and ventricular fibrillation (VF) or asystole occurs late in the process.^6^, ^7^

During the early stages of respiratory insufficiency, before cardiac arrest occurs, assisted ventilations may be all that is needed for survival. Following cardiac arrest, assisted ventilations and chest compressions must be added. In both cases, early CPR can be lifesaving.

## Survival of primary cardiac arrests was unchanged for decades

In spite of the American Heart Association's “Standards” in 1974, “Standards and Guidelines” in 1980, “Guidelines” in 1986, and “Updates of Guidelines” in 1992, 2000, and 2005, the survival rate of patients with out‐of‐hospital cardiac arrest (OHCA) in the USA averaged 7.6 percent and was unchanged from 1978 to 2008.^8^ In the USA, the reported survival of patients with OHCA secondary to VF, those most likely to survive, was also unchanged for decades, averaging 17.7%.^9^

The concept of the “chain of survival” for cardiopulmonary resuscitation published by Cummins and associates in 1991 was a major contribution to resuscitation science.^10^ However, any chain is only as strong as its weakest link. And the weakest link in the “chain of survival” was the rarity of bystander CPR. The incidence of bystander CPR was 32% in New York,^11^ 21% in Detroit,^12^ 15% in Ontario, Canada,^13^ 28% in Japan,^14^ 25% in Singapore,^15^ and 25% in the US CARES Registry in 2009^16^ and 28.6% in 2012.^8^ Only approximately one in four patients with witnessed OHCA was receiving bystander CPR.

A major reason for the low rate of bystander CPR was the decades‐old requirement of mouth‐to‐mouth ventilation (MTM) as the first step in bystander CPR. Because of the concerns of providing MTM ventilations, especially to strangers, bystanders, including medical personnel, would often call the emergency dispatch number, but then await EMS arrival.^17^, ^18^, ^19^ Such patients rarely survived.

But aversion to MTM ventilation is not the only reason for the less than optimal incidence of bystander CPR. A survey in Arizona in 2009, that we funded, found that the reasons for bystander's reluctance to provide CPR were almost equally divided among fear and concern of: (i) MTM contact, (ii) harming the person, (iii) legal consequences, (iv) not performing CPR properly, (v) being physically unable to perform CPR.^20^ These concerns must be addressed to increase the incidence of bystander CPR.

## Resuscitation research led to new approaches

Since the early 1990s, our University of Arizona Sarver Heart Center Resuscitation Research Group recommended “chest compression only CPR” (CO‐CPR) for witnessed primary cardiac arrest. In our animal models of VF arrest, we initially found that survival was similar with CO‐CPR and guideline protocols that recommended ventilations.^21^, ^22^ Support in humans for CO‐CPR at that time was a study by Hallstrom and associates of dispatcher‐assisted CPR that found CO‐CPR was as effective as standard CPR.^23^ Further support came later by the important SOS‐KANTO study from Japan.^14^

## Recognition of primary cardiac arrest

What is a primary cardiac arrest? It should be defined as “an unexpected, witnessed (seen or heard) collapse of a person who is not responsive and is not breathing normally.” This definition includes “and is not breathing normally” because the true frequency and nature of breathing followed by “gasping” from a primary cardiac arrest is not generally appreciated.^24^ This fact must be an integral part of our teaching to enhance the prompt recognition of primary cardiac arrest.

### Continued breathing after primary cardiac arrest

Following VF‐induced primary cardiac arrest, we found in swine that they continued to breathe normally for the first minute.^25^ To the author's knowledge, this phenomenon has not yet been documented in patients; therefore, it is an opportunity for the reader to do so. For those working in a sophisticated intensive care unit where both the patient's electrocardiogram and ventilations are monitored, there is a unique research opportunity.

### Gasping after primary cardiac arrest

In our resuscitation research laboratory, we found that, following the induction of primary VF in swine, breathing continued for about a minute. Gasping typically began in the second minute post arrest and followed a low frequency crescendo‐decrescendo pattern that stopped six minutes post arrest.^25^ Clark and associates reported gasping to be present in 55% of patients with witnessed OHCA.^26^ The prognosis of patients with OHCA is much better if gasping is present. In the state of Arizona, we found that 39% of patients with OHCA who were gasping on EMS arrival survived, compared with only 9% of patients who were not gasping.^27^

Therefore, to improve survival of patients with OHCA, the importance of, and the recognition of, gasping as a sign of a recent primary cardiac arrest must be taught and emphasized. Of course, if an automated external defibrillator is readily available, any bystander of a witnessed primary cardiac arrest should get the automated external defibrillator or order someone else to get it so it can be used if appropriate.^14^, ^28^

## Bystander CO‐CPR improved survival of animals with VF arrest

Our University of Arizona CPR Resuscitation Research Group published six studies with a total of 169 swine with variable durations of VF arrest before the initiation of basic life support with: (i) CO‐CPR, (ii) guidelines CPR with ventilations delivered over a 4‐s period, or (iii) no CPR for several minutes to simulate the lack of bystander CPR and the late arrival of EMS.^29^ Survival was 73% with CO‐CPR, 70% with “ideal CPR”, and 7% with no CPR.^29^ Thus, given the general reluctance of bystanders to perform MTM CPR, we have recommended CO‐CPR for patients with primary cardiac arrest since the 1990s.^6^

## Landmark observation on bystander CPR

For decades the CPR Guidelines rested on the assumption that “two quick breaths” would interrupt each set of chest compressions for only 4 s. However, a landmark observation published in 2000 showed that laypeople did not achieve anywhere near this speed when performing CPR. Resuscitation researchers from England made a critically important observation while Karl B. Kern M.D. of our resuscitation group was a visiting professor. These internationally recognized authorities taught the then “Guidelines” bystander CPR to a group of lay individuals. At the conclusion of the session, they recorded videos of some of these recently CPR certified lay individuals performing bystander CPR on mannequins. On review of these videos, they were surprised to find that these lay individuals interrupted chest compressions for an average of 16 s to deliver the then “Guidelines” recommended “two quick breaths” between each set of chest compressions.^30^

If lay bystanders did not achieve the Guidelines’ 4‐s interruption for ventilation, the question remained how quickly could more experienced performers of CPR interrupt chest compressions for the so‐called “two quick breaths”? We subsequently found that young healthy medical students interrupted chest compressions an average of 13 s and professional paramedics an average of 10 s to deliver the so‐called “two quick breaths”.^31^, ^32^

## Cerebral perfusion during bystander chest compressions for cardiac arrest

It took us years of laboratory research to discover the importance of CO‐CPR for myocardial perfusion and survival, but it took me listening to one recording of an emergency dispatcher's conversation between a wife receiving telephone CPR instructions to appreciate the deleterious effects of even short interruptions of chest compressions to cerebral perfusion. In that recorded EMS emergency call it must have taken some time before the paramedics arrived, as the wife eventually asked the phone dispatcher, “Why is it every time I press on his chest he opens his eyes, and every time I stop and breathe for him he goes back to sleep?”^33^ Obviously blood flow to the arrested individual's brain became inadequate soon after chest compressions were interrupted for MTM ventilations.

## New understanding of the pathophysiology of primary cardiac arrest

**Figure 2:**
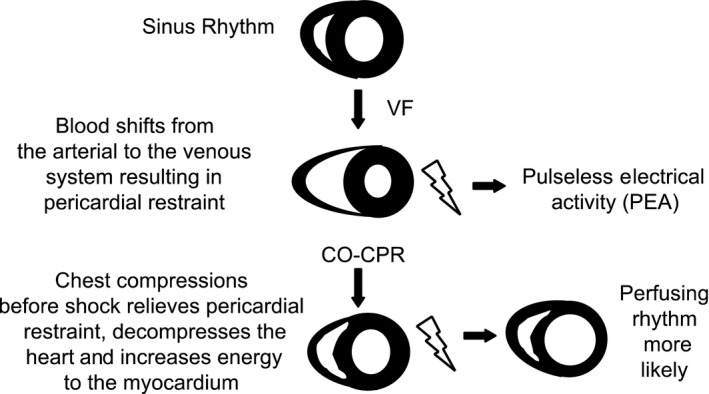
Cardiac blood volume shifts following ventricular fibrillation (VF) arrest and cardiopulmonary resuscitation. Following VF arrest, blood from the high‐pressure arterial system shifts to the low‐pressure venous system, resulting a decreased left ventricular volume but a marked increase in right ventricular volume, and in pericardial restraint. Application of chest compressions decreases pericardial restraint, perfuses the heart and brain, and increases the chances of a perfusing rhythm following defibrillation. CO‐CPR, chest compression only CPR; CPR, cardiopulmonary resuscitation.

We learned an incredible amount about the pathophysiology of primary VF arrest and resuscitation in 2002 during our visit to the research laboratory of Stig Steen, M.D., Ph.D. in Lund, Sweden.^34^ He reported that “In this pig model, VF caused venous congestion, an empty left heart, and a greatly distended right heart within 3 min.” His laboratory found that, during the first few minutes of VF arrest, the blood shifts from the higher‐pressure arterial system to the lower‐pressure venous system, resulting in a decrease in left ventricular (LV) volume and a marked increase in right ventricular (RV) volume (Fig. 2).^35^ This marked increase in the volume of the thin‐walled right ventricle resulted in pericardial constriction, and the decreased in LV volume contributed to pulseless electrical activity that is often observed following late defibrillation.

These volume shifts were shown by Steen in open chest swine, so we evaluated ventricular volume shifts in an intact chest of a swine by magnetic resonance imaging.^36^ We confirmed the marked increase in RV volume following cardiac arrest, which could contribute to pericardial constriction and cardiac tamponade following untreated cardiac arrest.^36^

## Chest compression only for primary cardiac arrest advocated

Based on our, Dr. Steen's, and others’ resuscitation research for primary cardiac arrest, we concluded in 2002 that we could no longer in good conscience continue to follow the American Heart Association guidelines for CPR and emergency cardiac care in Tucson. We announced our intentions and explained our rationale.^29^, ^37^, ^38^

In 2004, in cooperation with Bentley Bobrow, M.D., Medical Director, Bureau of EMS and Trauma System, Arizona Department of Health Services, we began a near state‐wide campaign to advocate CO‐CPR for primary cardiac arrest. The campaign included education with inserts in utility bills sent to households, free CO‐CPR training for the public carried out by our medical students and by some of Arizona's paramedics, training kits developed by Dr. Bobrow and colleagues that were sent to most junior high and high school students in public schools, by news stories, radio and television infomercials, and by celebrity endorsements.^39^ We also developed a short video for lay individuals describing CO‐CPR that by 2016 had more than 10 million “hits” (http://heart.arizona.edu/cpr-video).

**Figure 3:**
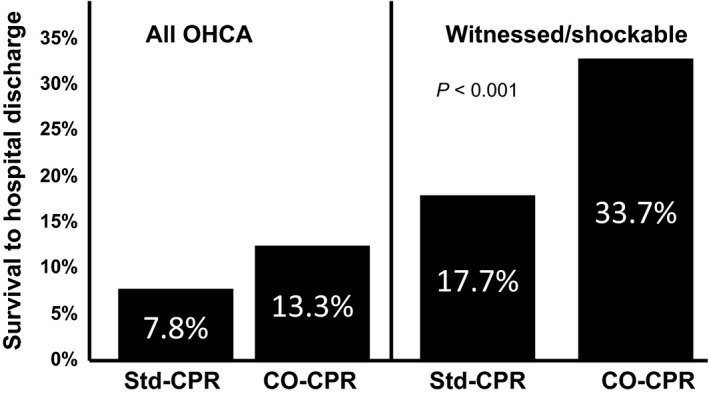
Survival of patients with OHCA in Arizona before and after bystander CO‐CPR was taught and advocated.

The results of our efforts to advocate and teach CO‐CPR for patients with primary cardiac arrest in the state of Arizona were published in 2010 (Fig. 2).^40^ The percentage of lay individuals who carried out CO‐CPR in Arizona dramatically increased from approximately 20% in 2006 to approximately 45% in the years 2007 and 2008, and to approximately 75% during 2008 and 2009.^40^ As shown in Figure 3, in the subset of patients with a witnessed cardiac arrest and a shockable rhythm, the survival rate was 34% in those receiving CO‐CPR and 18% in those receiving the then Guidelines CPR.^40^ Survival of patients with non‐cardiac arrest was 2.7% in those receiving CO‐CPR versus 3.8% in those receiving the then Guidelines CPR.^41^

The 7.8% survival of all patients with OHCA in this study in Arizona who received Guidelines CPR was not significantly different from the survival rate of 7.6% for all patients treated with Guidelines CPR with OHCA between 1950 and 2008, reported by Sasson and associates.^8^ The survival rate of patients with a witnessed OHCA and a shockable rhythm of 17.7% in those treated with Guidelines CPR was the same as the overall survival rate of patients with OHCA secondary to VF arrest in the USA, reported by Rea and associates.^8^, ^9^

Riva and associates recently reported that the increase in bystander CPR in Sweden during the last 15 years was mainly attributed to increased rates of CO‐CPR.^42^

## Early assisted ventilation not necessary for primary cardiac arrest

A major concern of CO‐CPR for primary cardiac arrest was the lack of assisted ventilation. Our colleague, Mathias Zuercher, M.D., an academic anesthesiologist from Switzerland, has, over the years, spent his vacations and his sabbatical working with our Resuscitation Research Group in Tucson. In an experiment of VF arrest, arterial blood gasses were measured at baseline, twice during CO‐CPR and passive oxygenation, and twice during assisted ventilation. During sinus rhythm, the arterial blood gas was 85 mmHg or 97% saturation, and fell to only 70 mmHg or 93% saturation following 9.5 min of untreated VF. The reason for the relative stability of the arterial blood saturation was that, following primary cardiac arrest, the blood remaining in the arterial system was still adequately saturated with oxygen. However, following the initiation of CO‐CPR, the arterial blood saturation decreased to 44 mmHg or 61% saturation after 14 min, and then to 31 mmHg or 34% saturation 16 min after primary cardiac arrest.^5^ The question arose, is this low arterial oxygenation harmful? Insight into this question was found in research on individuals climbing Mount Everest. In a report, one such individual had an arterial saturation of 34%.^43^ Thus saturations as low as 34% for long periods of time are compatible with life in humans.^43^

In Arizona, we found that the subset of patients with a witnessed OHCA and a shockable rhythm treated by CO‐CPR had a better survival rate than those treated with the then Guidelines CPR that recommended MTM ventilations that not only delayed the onset of chest compressions, but also resulted in their frequent and prolonged interruption.^39^ Following a near state‐wide advocacy for CO‐CPR for primary cardiac arrest in Arizona, we found that survival rose to 33.7% in those treated with bystander CO‐CPR compared with 17.7% in the subset treated with the then Guidelines CPR.^39^ Of interest was that, in our study, the survival rate of 17.7% in those with VF arrest treated with Guidelines CPR and ECC was exactly the same as the 17.7% survival rate in the USA between 1980 and 2003, reported by Rea and associates.^9^

## Cardiocerebral resuscitation changed EMS protocols

**Figure 4:**
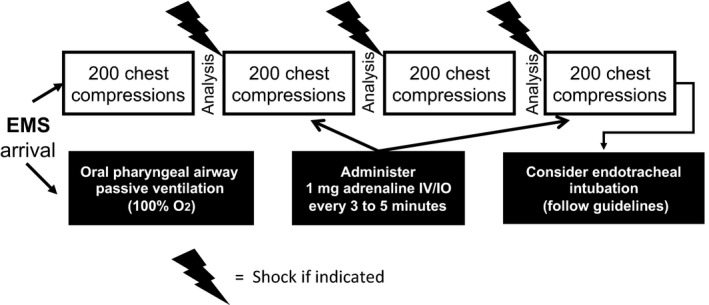
Pre‐hospital cardiocerebral resuscitation protocol for emergency medical services (EMS) first initiated in Tucson (AZ, USA).

Cardiocerebral resuscitation also significantly changed the protocol for EMS (Fig. 4). We did not allow urgent endotracheal intubation but rather advocated the prompt initiation of 200 uninterrupted chest compressions before and immediately after an indicated single defibrillator shock. A second EMS was to apply an oral pharyngeal airway with high‐flow oxygen (passive ventilation).^29^ Endotracheal intubation was delayed because of our observations of the frequent delay and prolonged interruptions of chest compressions by anesthesiologists during in‐hospital cardiac arrests. We assumed that EMS must also not infrequently experience difficulty. Subsequent studies by Wang and associates confirmed this assumption.^44^ As shown in Figure 3, this sequence of continuous chest compressions before and after a defibrillation was repeated until a perfusing rhythm is established or a series of four sets of continuous chest compressions have been completed.^29^ Another component was early adrenaline administration.^29^, ^38^

## Positive pressure ventilation can be harmful during resuscitation

Are there other benefits of passive ventilation during resuscitation of individuals following primary cardiac arrest? Dr. Thomas Aufderheide and associates made major contributions to CPR research, including their finding that hyperventilation during resuscitation efforts by EMS were common. They reported that the excessive frequency of positive pressure ventilations were harmful during resuscitation efforts, as they caused excessive increases in intrathoracic pressures, thereby decreasing venous return to the thorax and thus subsequent cardiac output.^45^, ^46^, ^47^

## Cardiocerebral resuscitation improved survival

**Figure 5:**
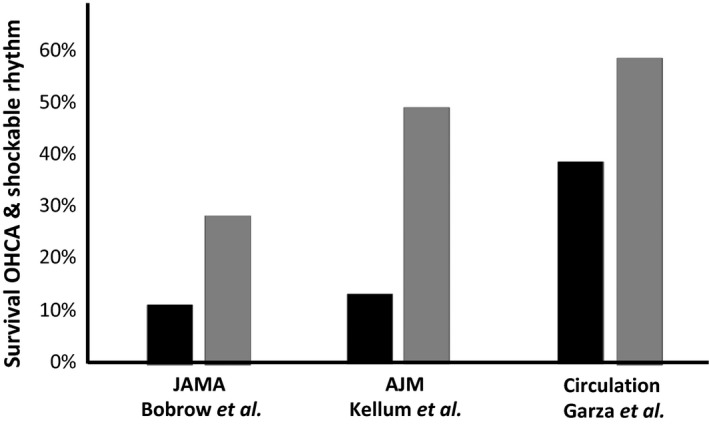
Survival of patients with out‐of‐hospital cardiac arrest (OHCA) and a shockable rhythm improved in each region where cardiocerebral resuscitation emergency medical services protocols were instituted. The dark columns indicate the percent survival of patients with out‐of‐hospital cardiac arrest (OHCA) and a shockable rhythm when the paramedics arrived when the paramedics were followed the then American Heart Association (AHA) Guidelines for Emergency Medical Services (EMS). The gray columns indicate percent survival in each of these areas following the EMS institution Cardiocerebral Resuscitation.

The “community” component of CCR has been shown to improve survival in Arizona, and the “pre‐hospital” components of CCR improved survival (Fig. 5) in each area in which it was instituted; first in Rock and Walworth counties (WI, USA), in Kansas City (MO, USA), and in Arizona.^39^, ^48^, ^49^, ^50^

A systemic review and meta‐analysis reported that, compared with older guidelines, CCR was associated with a significant survival benefit, including a threefold increase in survival for patients with witnessed ventricular fibrillation arrest.^51^

The “hospital” component of CCR (Fig. 1), added a few years later, has also been shown in Arizona to be independently associated with increased overall survival and favorable neurological outcome.^52^

## Online videos of cardiocerebral resuscitation

Online videos teaching and explaining bystander CO‐CPR for the lay public and the science behind the EMS response of CCR for paramedics are available at http://heart.arizona.edu/learn-cpr.

## Alternatives to following guidelines

There are a number of limitations to the Guidelines process for CPR and EMS therapy of cardiac arrest that the author articulated in 2009.^52^ In addition, there are dramatically different survival rates among different EMS following the same Guidelines.^53^ It would be nice to have randomized controlled studies in humans for all Guidelines changes, but this has proved to be nearly impossible. Therefore, an alternative approach to improving survival is to document your results, to make reasonable changes based on research, and to determine if the changes improved survival.^54^

## Conclusions

Cardiocerebral resuscitation is a new approach to the management of patients with primary OHCA that has been shown to significantly improve survival of patients with VF arrest. It is critically import that each EMS unit knows the survival rate of its patients with primary cardiac arrest and a shockable rhythm, the subgroup most likely to survive, as it varies greatly among different areas. For cardiac arrest secondary to respiratory insufficiency, such as drowning or drug overdose, the current Guidelines for Cardiopulmonary Resuscitation (bystander ventilations plus chest compressions) are indicated.

## Disclosure

Conflict of Interest: None declared.
